# Motion Detection and Correction for Frame-Based Stereotactic Localization

**DOI:** 10.7759/cureus.28387

**Published:** 2022-08-25

**Authors:** Mark Sedrak, Patrick Pezeshkian, David Purger, Siddharth Srivastava, Ross Anderson, Derek W Yecies, Elena Call, Suketu Khandhar, Keegan Balster, Ivan Bernstein, Diana M Bruce, Armando L Alaminos-Bouza

**Affiliations:** 1 Neurosurgery, Northern California Kaiser Permanente, Redwood City, USA; 2 Neurological Surgery, Stanford University Hospital, Palo Alto, USA; 3 Neurological Surgery, Kaiser Permanente Redwood City, Redwood City, USA; 4 Neurosurgery, Stanford University, Stanford, USA; 5 Neurosurgery, Kaiser Permanente Redwood City, Redwood City, USA; 6 Neurology, Kaiser Permanente Redwood City, Redwood City, USA; 7 Neurology, Kaiser Permanente Sacramento, Sacramento, USA; 8 Research, Kaiser Permanente Redwood City, Redwood City, USA; 9 Medical Physics, MEVIS Informática Médica Ltda., São Paulo, BRA

**Keywords:** stereotactic and functional neurosurgery, stereotactic and functional, cartesian coordinate system, preoperative localization, stereotactic frame

## Abstract

Frame-based stereotactic localization is an important step for targeting during a surgical procedure. The motion may cause artifacts in this step reducing the accuracy of surgical targeting. While modeling of motion in real-life scenarios may be difficult, herein we analyzed the case where motion was suspected to impact the localization step. In this case, a scan with and without motion was performed with a 3N localizer, allowing for a thorough analysis. Pseudo-bending of straight rods was seen when analyzing the data. This pseudo-bending appears to occur because head-frame motion during imaging acquisition decreases the accuracy of the subsequent reconstruction, which depends on Digital Imaging and Communications in Medicine (DICOM) metadata to specify the slice-to-slice location that assumes embedded object stability. Comparison of single-slice and multi-slice stereotactic localization allowed for comparative errors for each slice in a volume. This comparative error demonstrated low error when the patient was under general anesthesia and presumed not to have moved, whereas a higher error was present during the scan with motion. Pseudo-bending can be corrected by using only localizer fiducial-based information to reorient the pixels in the volume, thus creating a reoriented localizer scan. Finally, targeting demonstrated a low error of 0.1 mm (+/- 0.1 mm) using this reoriented localizer scan, signifying that this method could be used to improve or recover from motion problems. Finally, it is concluded that stability and elimination of motion for all images utilized for stereotactic surgery are critical to ensure the best possible accuracy for the procedure.

## Introduction

Frame-based stereotactic localization involves the detection of fiducials that are typically created by 3 N-localizers in a set of medical images to develop a surgical targeting coordinate system [1]. The fiducials in the images are generated from bars of known geometry attached to a head-mounted stereotactic frame that is imaged along with the patient. In practice, frame-based localization can be subject to errors that may impact surgical accuracy. As a result, error minimization is an important adjunct to stereotactic localization that obtains the best accuracy to improve the result of surgery [2]. Some factors that can impact localization accuracy include resolution of the imaging technique, imperfect detection of the centroids of the fiducials in the image, or motion of the patient during the imaging acquisition. While the first two are not the focus of this report, the motion may impact stereotactic localization accuracy. Therefore, motion detection is fundamental to quality assurance. Comparison between single-slice and multi-slice localization may add clarity regarding the degree of motion [3]. Herein, we describe a case where we observed errors between single-slice and multi-slice localization techniques which could be ascribed to motion. 

## Case presentation

**Figure 1:**
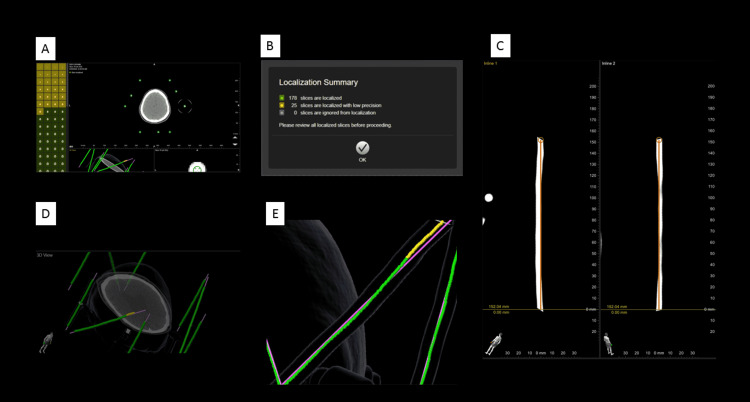
Stereotactic localization demonstrating low precision (yellow) of 25 of the 203 slices (A, B). Placing a trajectory along the rods demonstrates the pseudo-bending more clearly (C). Finally, the imaging software allows for direct visualization of an expected geometry (purple lines) and the deviation (yellow) that corresponds to the low precision slices (D, E). Image Capture from Brainlab Elements Stereotaxy (Brainlab Inc, Feldkirchen, Germany)

A 73-year-old individual with Parkinson's Disease (PD) with severe right-sided tremor predominant symptoms was treated via left subthalamic nucleus (STN) deep brain stimulation (DBS). Preoperatively, a magnetic resonance image (MRI) was obtained for surgical target planning. On the day of surgery, a planned awake DBS procedure was planned and the stereotactic frame and localizer were attached followed by computed tomography (CT) image series obtained using a diagnostic CT scanner (General Electric 64 slice Discovery HD750 CT Scanner, 0.625 mm slice thickness and spacing between slices, 120 kVp, 512 Rows, 512 Columns, 0.781250\0.781250 Pixel Spacing). The image sequence contained 203 images which we describe as the "bad scan" and was processed by stereotactic planning software, Brainlab Elements Stereotaxy (Brainlab Inc, Feldkirchen, Germany), for image fusion, localization, and the surgical coordinate calculation. During the localization step for the BRW Localizer Frame (BRWLF) (Cosman-Roberts-Wells/Brown-Roberts-Wells Localizer Frame, Radionics CRW Stereotactic System, Integra LifeSciences Corporation, Plainsboro, New Jersey), 25 of the 203 images were localized with low precision (Figure 1). This high percentage of low-precision images was due to the patient's severe tremors and patient intolerance of the scanning procedure. In consequence, the patient was placed under general anesthesia and the localizer CT scan was repeated under these conditions. This second localization CT series, which was initially unplanned, demonstrated excellent localization of the 217 slices, which we describe as the "good scan".

**Figure 2:**
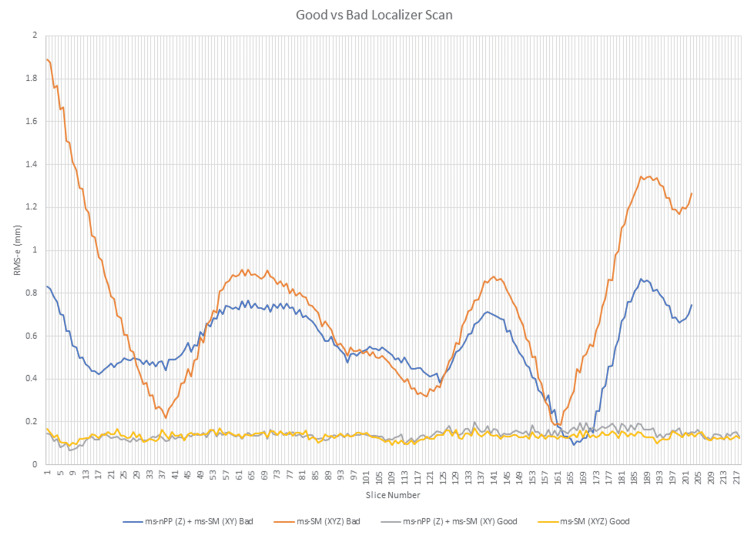
Analysis of good versus bad scan sequences. Herein, we have analyzed single-slice versus multi-slice methods to generate a root mean square error (RMS-e) for all nine fiducial rods on a slice-by-slice basis. When using multi-slice methods, there are two options: multi-slice normal to parallel planes (ms-nPP) for the z-axis and multi-slice stereotactic matrix (ms-SM) for the x- and y-axes or ms-SM for the x-, y-, and z-axes. Comparative data are displayed confirming that the bad scan sequence contained higher RMS-e than the good scan sequence, which appeared more stable with less oscillation. The data demonstrate oscillation in the bad scan sequence but not in the good scan sequence. This oscillation is presumably related primarily to head-frame motion. Graph generated from Excel (Microsoft Corporation, Redmond, Washington)

The good and bad scans provided two sets of images for the same patient using a 3 N-localizer apparatus and without altering the apparatus between scans, thus allowing a thorough offline analysis. Previously published single-slice and multi-slice stereotactic localization techniques were used to analyze the scan data [3]. Single-slice stereotactic localization utilizes only a single image for a set of fiducials, whereas multi-slice stereotactic localization can utilize the entire set of images in a sequence, but then be used to compute data on a per slice basis. The results of these two localization techniques can then be compared to detect errors that can, in part, be ascribed to motion. An analysis of the good and bad CT scan sequences was performed via in-house custom software (available on request) to numerically analyze such errors for each study, as follows. In each slice, the nine three-dimensional (3D) rod positions were computed using single-slice and multi-slice methods and the root means square error (RMS-e) was computed using those data (Figure 2). Two localization options exist for multi-slice data; these options are multi-slice normal to parallel planes (ms-nPP) for the z-axis and multi-slice stereotactic matrix (ms-SM) for the x- and y-axes or ms-SM for the x-, y-, and z-axes. The data demonstrate high error with an apparent oscillation in the bad scan sequence but demonstrate relatively low error in the good scan sequence. This oscillation is presumed to be related to head-frame motion during the imaging acquisition. Interestingly, ms-nPP generally reduced the error for the bad scan over ms-SM. 

**Figure 3:**
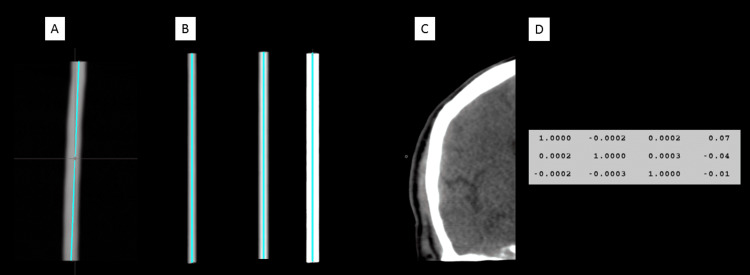
Comparison study between good and bad scan sequences. As previously noted, pseudo-bending of a rod (A) is observed due to the reconstruction of the pixels in multiple slices using only DICOM metadata, which assumes imaging-object stability. However, given that each individual image contains a 3 N-localizer system, we can reorient the pixels in the imaging volume, ignoring the DICOM metadata. Using only this 3 N-localizer information, several rods demonstrate a perfectly straight reconstruction (B) adjacent to a brain/skull image (C). Finally, we can take the bad scan sequence with reoriented pixels and then fuse this with the good scan sequence. The fusion result demonstrates a nearly perfect 3 x 4 identity matrix using mutual information, suggesting that the reoriented pixels from the bad scan sequence matched the pixels from the good scan sequence (D). Digital Imaging and Communications in Medicine (DICOM). Images from MNPS - Mevis Informatica Medica, São Paulo, Brazil

Next, the option of using only single-slice data to reconstruct and reorient the pixels in the imaging volume was explored. As mentioned in Figure 1, pseudo-bending of the rods visibly confirms a problem with the bad scan sequence. This pseudo-bending reconstruction can be considered to be reconstruction from the Digital Imaging and Communications in Medicine (DICOM) imaging coordinate, which assumes stability of imaging-object systems. Correction of this pseudo-bending is made possible by the fiducials present in the images (Figure 3). Pseudo-bending is present primarily because head-frame motion likely occurred during imaging acquisition (Figure 3A). However, given that the 3 N-localizers permit computation of a three-dimensional (3D) plane for each slice, we can reorient the pixels in the imaging volume, ignoring the DICOM coordinate system and metadata. When using only the 3 N-localizers to direct the reorientation of the pixels in the imaging volume, we demonstrate a perfectly straight reconstruction of several rods (Figure 3B) adjacent to a clear brain/skull image (Figure 3C). Finally, we can take the bad scan with reoriented pixels, again using only the 3 N-localizers, and then fuse this with the good scan demonstrating a nearly perfect identity matrix using mutual information (MI) (Figure 3D). 

Finally, various target coordinates from the patient's preoperative MRI were fused to the reoriented pixels from the good and bad CT scan sequences. The frame-based stereotactic CRW (Cosman-Roberts-Wells/Brown-Roberts-Wells Localizer Frame, Radionics CRW Stereotactic System, Integra LifeSciences Corporation, Plainsboro, New Jersey) coordinates were then compared between these two sets (Table 1). Errors were low between the two sequences, approximately 0.1 mm (+/- 0.1 mm). This result demonstrates that the bad scan sequence with reoriented pixels could potentially be used for the surgical procedure to achieve good accuracy. 

**Table 1:** Preoperative Magnetic Resonance Imaging (MRI) with stereotactic targets fused to good Computed Tomography (CT) localizer scan versus reoriented pixels of bad CT localizer scan to generate stereotactic coordinates Stereotactic coordinates are computed and compared in stereotactic space. The errors in lateral (Lat), anteroposterior (AP), and vertical (Vert) average 0.1 mm (+/- 0.1 mm). Given the low errors between the two studies, the data suggest that the bad scan sequence with reoriented pixels could have been used for the surgical procedure. However, more studies and analyses of numerous patients are needed to validate this hypothesis. AC - Anterior Commissure; PC- Posterior Commissure; IHP - Interhemispheric point; VIM-L - Ventral Intermediate Nucleus of the Thalamus on the left; VIM-R - Ventral Intermediate Nucleus of the Thalamus on the right; ZI-L - Zona Incerta on the left; ZI-R - Zona Incerta on the right; GPI-L - Globus Pallidus Interna on the left; GPI-R - Globus Pallidus Interna on the right

	GOOD CT				BAD CT				Error			
	Lat	AP	Vert		Lat	AP	Vert		Lat	AP	Vert	
AC	0.12	12.56	-22.05		0.14	12.74	-22.05		-0.02	-0.18	0	
PC	-1.67	-14.21	-19.67		-1.62	-14.04	-19.69		-0.05	-0.17	0.02	
IHP	-0.93	-11.48	11.7		-0.66	-11.33	11.69		-0.27	-0.15	0.01	
VIM-L	-13.82	-4.97	-20.1		-13.78	-4.81	-20.02		-0.04	-0.16	-0.08	
VIM-R	12.18	-6.94	-20.57		12.22	-6.75	-20.67		-0.04	-0.19	0.1	
ZI-L	-11.85	-3.16	-23.03		-11.83	-2.99	-22.96		-0.02	-0.17	-0.07	
ZI-R	9.84	-4.74	-23.42		9.86	-4.55	-23.5		-0.02	-0.19	0.08	
GPI-L	-21.38	4.49	-26.53		-21.39	4.65	-26.39		0.01	-0.16	-0.14	
GPI-R	19.8	1.66	-27.27		19.79	1.86	-27.41		0.01	-0.2	0.14	Average
								Average	-0.04889	-0.17444	0.006667	-0.07222
								Standard Deviation	0.080615	0.015713	0.086281	0.06087

## Discussion

The target coordinates in frame-based stereotaxis are calculated via the culmination of multiple steps in the surgical planning process. These steps include direct and indirect target planning on magnetic resonance images (MRI), image fusion of different sequences (often MRI with computed tomography (CT)), and stereotactic localization [4-6]. Therefore, stereotactic localization is a critical step in the process that produces surgical coordinates. Also, stereotactic localization affords the opportunity for detailed study due to the embedded fiducials of known geometry in the images. 

Herein, we describe a unique case where stereotactic localization resulted in one bad and one good scan sequence that we could compare directly. In this analysis, we have demonstrated the utility of a 3 N-localizer system which allows the comparison of single-slice and multi-slice stereotactic localization [3]. Using this information, errors can be computed that can be the result of motion. Low errors and oscillations suggest a lack of head-frame motion, whereas high errors and oscillations suggest head-frame motion. It should be noted that other causes of image noise, such as poor centroid localization of a fiducial rod or image blurring, may also introduce errors. Identification of this head-frame motion can be detected by in-line reconstruction of known straight fiducial rods, creating what we have termed pseudo-bending. This pseudo-bending is the result of a reconstruction based on the assumed equal spacing between slices. However, because a 3 N-localizer system permits the computation of three 3D points within each slice, we are then able to compute a 3D plane for each slice that enables us to reorient all pixels in the volume. This method of reorienting the pixels was then demonstrated by straightening the fiducial rods to eliminate pseudo-bending, excellent overlap with the presumed good scan sequence, and minimal error associated with stereotactic targeting. 

**Figure 4:**
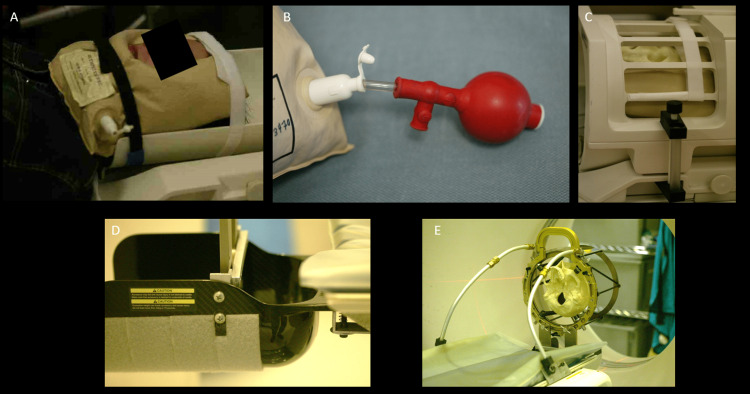
Methods of motion stabilization during MRI and CT imaging. In the absence of rigid skull fixation such as for a preoperative MRI, an MRI-compatible bean bag can be used to stabilize the head (A). A suction tool can be used to remove air from the bean bag which is affixed to the patient (B). This then can fit within the parameters of the MRI coil (C). When the head is fixated in a stereotactic apparatus, such as for a CT scan sequence for stereotactic localization, a posterior bracket attached to the stereotactic frame can be used to support the head (D). If further fixation is needed, rod brackets can be used to support the stereotactic frame anteriorly (E). These rod brackets (E) are not routinely used in our center, but the posterior bracket (D) is used for all cases, such as for the good and bad image sequences. Head-frame motion can still occur during imaging acquisition despite these methods to reduce motion and careful analysis is needed for all the final images in each sequence. MRI - Magnetic Resonance Imaging, CT - Computed Tomography, Images courtesy of Eric Sabelman, PhD and Gary Heit, PhD, MD

Although this case report discusses a 3 N-localizer system, any system with greater than 3 N-localizers could also have been conceivably used to compute a 3D plane via an overdetermined method [2, 7]. In contrast, a 2 N-localizer system fails to provide enough information to compute a 3D plane using diagonal bar positions. Elements that could not be controlled in this analysis include the absolute resolution of the CT scanner, motion/artifact from MRI, and image fusion errors. However, the data suggest that motion must be carefully controlled during preoperative imaging. While a localizer CT scan sequence clearly allows for a reorientation of pixels, sequences that do not include fiducials cannot be reoriented. For example, in this case, the preoperative MRI did not contain rod fiducials and therefore motion artifacts could not be controlled. In our center, we use rigid fixation of the head for all CT and MRI imaging related to stereotaxis using an MRI-compatible bean bag as well as bracket fixation of the stereotactic frame during CT imaging (Figure 4). Nevertheless, the problem of head-frame motion requires careful attention even when these techniques are applied. Lastly, software solutions may allow for motion correction in circumstances where sufficient fiducials are present in the individual images.

## Conclusions

Stereotactic localization is a critical step for frame-based stereotactic procedures and motion during the imaging acquisition may lead to inaccuracy. Head-frame systems that contain sufficient fiducials, such as a 3 N-localizer, allow for analysis between single-slice and multi-slice localization. This comparison can determine the stability or motion for the imaging sequence. When sufficient data are present, pixel reorientation may serve a role in correcting data obtained in the presence of patient motion. These ideas merit further exploration as they may lead to improved accuracy.
